# One population, many risks: rethinking youth suicide prevention in the Americas

**DOI:** 10.1016/j.lana.2026.101608

**Published:** 2026-08-14

**Authors:** The Lancet Regional Health – Americas

**Keywords:** **Editorial Disclaimer:** This editorial discusses suicide and mental health affecting youth.

In the Americas, suicide rates have moved in the opposite direction to the rest of the world. Between 2000 and 2021, the region saw a 17% increase in age-standardised suicide mortality, while the global rate decreased by approximately 35% over the same period. This divergence reflects deep structural and social heterogeneity across the continent. According to PAHO, in North America, the USA and Canada record the highest sub-regional suicide average, at 13.5 per 100,000 population. While the Andean region reports rates of 5.0 per 100,000, parts of South America are home to the continent's highest rates: over 22 per 100,000 in Guyana, Suriname, and Uruguay. Population-level trends, however, can obscure critical vulnerabilities within specific subgroups. A study examining individuals with autism in Manitoba, Canada, found that females face a higher suicide risk than males, a reversal of the general sex pattern in suicide, underscoring the value of disaggregated data. Prevention strategies must therefore identify the groups and settings where risk accumulates. Among these, young people are the central concern of this discussion.

Suicide is one of the leading causes of death in young people in the Americas, and mortality related to suicide has increased over the past 20 years. This population faces rapid change, social pressure, and bullying across multiple settings, all of which can precipitate crises requiring early, sustained attention. The increase has been greater among young females than males, and it is alarming that the sharpest rise was recorded among children aged 10–14 years, an age at which suicide had been considered rare. GBD estimates from Brazil show that self-harm is already the eighth contributor to premature death at ages 10–14 years, and a study including a Brazilian cohort of 2511 children aged 6–14 years concluded that female sex and childhood trauma are associated with an earlier onset and a greater number of suicide attempts, underscoring the need for cross-sector prevention efforts that include suicidal ideation and other early signs.

Among the environments shaping adolescent mental health, none has emerged more rapidly than the digital space. Social media platforms expose young people to curated lives that bear little resemblance to reality, and this uncontrolled access has been linked to depression, anxiety, and suicidal ideation, particularly among girls. The evidence is still emerging, but that is no reason to postpone proportionate action. The harms of excessive digital exposure have begun to prompt regulatory responses. Platforms now offer supervised modes for adolescents, devices embedded with screen-time controls, and several countries restrict phones in schools, though evidence on the effect of these restrictions is very recent and far from conclusive. But the internet is not only a threat. Many young people access mental health resources, crisis lines, and peer support through digital channels, spaces that might represent their only point of contact. Yet the quality of these channels is often unverifiable. The challenge is how to shape digital engagement, ensuring platforms are designed with adolescent safety as a non-negotiable feature from the start.

Prevention must reach young people where they already are, and schools are key. Although they can also be a source of distress, schools can offer pedagogical and psychological support, equip staff to recognise warning signs and act as an early bridge to care. Evidence for well-implemented school mental health programmes is growing, and some countries have gone further with broad, low-barrier youth services; Australia's headspace network is an example. Schools, however, can do more than just connect. An upcoming Lancet Psychiatry Commission is set to discuss that schools must be placed at the centre of how we conceptualise and deliver public mental health for adolescents, with a clear focus on having their voices included at all stages. But schools alone cannot reach everyone, and for most people at risk, mental health services are scarce and concentrated in wealthier settings, so the health system's first, and often only, point of contact is primary care. Embedding mental health into primary care means that someone in distress can be recognised and supported earlier by a trained primary care team, through routine screening, brief intervention, and follow-up, rather than referred into a specialist system they might never reach.

The rise in suicide is a public health crisis, and for young people, it demands action shaped around their specific needs, exposures, and barriers to care. Reversing the Americas' trajectory of suicides requires progress on three interdependent fronts: stronger and more disaggregated surveillance, so prevention strategies can reach the populations most at risk; digital platform regulation that makes adolescent safety a design requirement; and integration of mental health into schools and primary care, so that response does not depend on specialist services that remain inaccessible to most. Preventing youth suicide will not come from doing more of the same, but from strategies shaped around and with young people themselves.

